# Ethnic differences in the comparative effectiveness of second‐line type 2 diabetes medications in preventing cardiovascular disease

**DOI:** 10.1111/dom.70273

**Published:** 2025-11-12

**Authors:** Mia Harley, Christopher T. Rentsch, Elizabeth Williamson, Anoop S. V. Shah, Patrick Bidulka, Charlotte Warren‐Gash, Marleen Bokern, Rohini Mathur

**Affiliations:** ^1^ Faculty of Epidemiology and Population Health London School of Hygiene & Tropical Medicine London UK; ^2^ Department of Internal Medicine Yale School of Medicine New Haven Connecticut USA; ^3^ Faculty of Public Health and Policy London School of Hygiene & Tropical Medicine London UK; ^4^ Wolfson Institute of Population Health Queen Mary University of London London UK

**Keywords:** cardiovascular disease, DPP‐4 inhibitor, real‐world evidence, SGLT2 inhibitor, sulphonylureas, type 2 diabetes

## Abstract

**Aim:**

To investigate ethnic differences in the comparative effectiveness of sulfonylureas (SU), dipeptidyl peptidase‐4 inhibitors (DPP4i) and sodium‐glucose cotransporter‐2 inhibitors (SGLT2i) on cardiovascular outcomes.

**Materials and Methods:**

We identified adults with type 2 diabetes in UK electronic health records initiating SU, DPP4i or SGLT2i (2015–2022). The outcomes were major adverse cardiovascular events (MACE: myocardial infarction, stroke, heart failure hospitalisation, cardiovascular death). Cox models estimated hazard ratios for DPP4i versus SU, SGLT2i versus SU and SGLT2i versus DPP4i. Wald tests assessed interaction by ethnicity.

**Results:**

Among 91 116 included individuals (72.3% White, 14.2% South Asian, 6.0% Black), 34.2% initiated an SU, 42.0% DPP4i and 23.8% SGLT2i. There was weak evidence of interaction by ethnicity for DPP4i versus SU on MACE (*p* = 0.12), with stronger effects observed for DPP4i in the Black group (hazard ratio [HR]: 0.64, 95% confidence interval [CI]: 0.46–0.89) than White (HR: 0.91, 95% CI: 0.84–0.98) or South Asian (HR: 0.93, 95% CI: 0.75–1.16) groups. There was evidence of interaction by ethnicity for DPP4i versus SU on heart failure hospitalisation (*p* = 0.05), with a stronger effect observed for DPP4i in the Black group (HR: 0.50, 95% CI: 0.30–0.84). There was no clear evidence of ethnic differences for other treatment comparators or cardiovascular outcomes.

**Conclusions:**

We found weak evidence suggesting a greater effect of DPP4i than SUs against MACE in Black people, particularly for heart failure hospitalisation, but no evidence of other ethnic differences in treatment effects.

## 1 INTRODUCTION

There are large ethnic inequalities in the development of cardiovascular complications among people with type 2 diabetes in the United Kingdom.^1^, ^2^, ^3^ One potential contributor to inequalities may be clinical guidelines, which are developed using data from predominantly White populations, potentially resulting in suboptimal care among non‐White ethnic groups.

While UK clinical guidelines for type 2 diabetes currently recommend several classes for second‐line treatment, there is limited guidance on which medications should be prescribed to whom.^4^ There are plausible biological and clinical explanations for potential ethnic differences in the cardiovascular effects of glucose‐lowering treatments. Ethnic variations in type 2 diabetes pathophysiology, including the role of insulin resistance and β‐cell function in diabetes,^5^, ^6^, ^7^ may influence the effectiveness of certain medication classes. There are also well‐established ethnic differences in baseline cardiovascular risk,^8^ which may extend to ethnic differences in the cardiovascular effects of treatments. People of South Asian descent have been found to have a higher risk of myocardial infarction, stroke and cardiovascular death, after adjustment for socioeconomic and clinical factors, compared to White and Black groups in the United Kingdom.^9^ African Americans have been shown to have higher rates of heart failure than other ethnic groups in the United States,^10^, ^11^, ^12^, ^13^, ^14^ indicating potential ethnic differences in heart failure aetiology. Nevertheless, ethnic differences in type 2 diabetes treatment effects have not been well explored.

Some cardiovascular outcome trials of glucose‐lowering medications have suggested ethnic differences in the treatment effects on certain cardiovascular outcomes,^15^, ^16^, ^17^ however, evidence has been inconsistent across trials.^18^, ^19^, ^20^, ^21^ Non‐White groups have often been underrepresented in clinical trial populations, which has limited the ability to detect ethnic differences in treatment effects. The generalisability of these clinical trial findings to real‐world settings is also unclear, as most are restricted to people with pre‐existing cardiovascular disease and compare medications to placebo, with inconsistent use of background glucose‐lowering therapy.

We aimed to address this gap by investigating ethnic differences in the comparative effectiveness of the three most prescribed second‐line type 2 diabetes medications, sulfonylureas (SU), dipeptidyl peptidase‐4 inhibitors (DPP4i) and sodium‐glucose cotransporter‐2 inhibitors (SGLT2i),^22^ in preventing cardiovascular outcomes using large UK real‐world data sources.

## 2 MATERIALS AND METHODS

### 2.1 Study design, setting and participants

We conducted an observational study using UK electronic health records from Clinical Practice Research Datalink (CPRD) Aurum, which covers approximately 25% of the current UK population.^23^ These data were linked to Hospital Episode Statistics (HES) admitted patient care data, Office for National Statistics (ONS) death records and index of multiple deprivation (IMD) patient‐level and practice‐level datasets.

The study period was 1 January 2015 to 28 February 2022, to align with National Institute for Health and Care Excellence (NICE) type 2 diabetes guidelines during which limited prescribing recommendations effectively resulted in ‘quasi‐random’ allocation of second‐line medication classes.^22^, ^24^

The inclusion criteria were adults with type 2 diabetes registered at a general practice in England who initiated treatment with a SU, DPP4i or SGLT2i during the study period, and had a metformin prescription within the 90 days prior to initiation. Individuals were excluded at baseline who had a record of any other antidiabetic medication, a glycated haemoglobin (HbA1c) measurement of <48 mmol/mol (6.5%) at baseline, pre‐existing cardiovascular conditions, less than 12 months of follow‐up or missing sex data (Table S1).

### 2.2 Exposure and outcome

**FIGURE 1:**
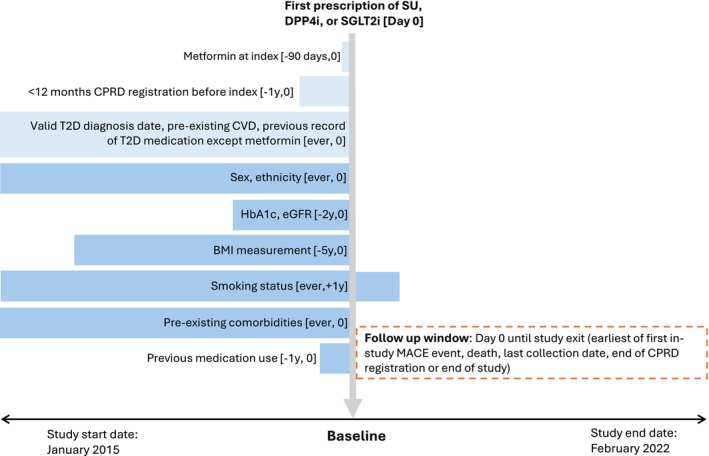
Timeline of inclusion, exclusion and covariate assessment relative to baseline. Baseline (Day 0) was defined as the date of first prescription of sulfonylureas (SU), dipeptidyl peptidase‐4 inhibitors (DPP4i) or sodium‐glucose cotransporter‐2 inhibitors (SGLT2i). Blue bars indicate the assessment windows for inclusion/exclusion criteria (light blue) and covariates (mid blue), while the dashed orange bar indicates the follow‐up period. Details of inclusion, exclusion and covariate definitions are provided in Tables S1 and S3. BMI, body mass index; CPRD, Clinical Practice Research Datalink; eGFR, estimated glomerular filtration rate; HbA1c, glycated haemoglobin; MACE, major adverse cardiovascular events.

Baseline was defined as the date of first prescription of SU, DPP4i or SGLT2i. Individuals remained in the exposure group to which they were assigned at baseline, irrespective of subsequent changes in treatment. Individuals were followed until the end of registration, the end of the practice contributing data to CPRD, the end of the study period (28th February 2022), non‐cardiovascular death or the occurrence of an outcome of interest, whichever occurred first (Figure 1). The primary outcome was major adverse cardiovascular events (MACE), a composite outcome including myocardial infarction, stroke, heart failure hospitalisation and cardiovascular death. These outcomes were chosen based on recent cardiovascular outcomes trials highlighting potential ethnic differences in treatment effects for these outcomes.^15^, ^16^, ^18^, ^19^, ^20^, ^25^, ^26^ The secondary outcomes were the individual cardiovascular components in MACE. Further details on outcome definitions are provided in Table S2.

### 2.3 Covariates

Potential confounders were identified through a review of existing literature and consideration of a directed acyclic graph (Figure S1). Data were extracted on the following at baseline: age, sex, ethnicity, IMD, region of England, calendar year, years since type 2 diabetes diagnosis, HbA1c, body mass index (BMI), estimated glomerular filtration rate (eGFR), smoking, history of alcohol misuse, rate of primary care consultations in the year prior, pre‐existing comorbidities (atrial fibrillation, peripheral artery disease, hypertension, neuropathy, retinopathy, all cancers, chronic liver disease, chronic respiratory disease, rheumatoid arthritis, dementia, severe mental illness and common mental disorders) and medication prescription in the year prior (angiotensin‐converting enzyme inhibitors [ACEis], angiotensin II receptor blockers [ARBs], calcium channel blockers, diuretics, statins, antiplatelets, anticoagulants and antipsychotics). Further details on covariate definitions can be found in Table S3.

### 2.4 Statistical analysis

Descriptive statistics were reported for each treatment group in the total study population and for each treatment group in the White, South Asian and Black groups. Descriptive statistics, incidence rates and hazard ratios (HRs) were not reported for the smaller ethnic groups (Mixed, Other and Not stated) due to small numbers. Crude incidence rates for outcomes were calculated for each treatment group in the total study population and for each treatment group in the White, South Asian and Black groups.

Multiple imputation was used to handle missing covariate data for time‐to‐event analyses, under the assumption that the data were missing at random. Multiple imputation by chained equations was used to generate 10 imputed datasets in each treatment group. Ethnicity, IMD and smoking status were imputed as categorical variables using multinomial logistic regression models. HbA1c, BMI and eGFR were imputed as continuous variables using linear regression models. The imputed dataset was used to estimate the association between treatment class and cardiovascular outcomes for the main analyses. ‘Not stated’ ethnicities were not imputed and instead retained as an ethnicity category in the analyses because these can arise when people are asked their ethnicity and choose not to provide the information.^27^ Therefore, we do not believe they fulfil the missing at random assumption.

Cox proportional hazards models were used to estimate HRs and 95% confidence intervals (CIs) for cardiovascular outcomes for: (i) DPP4i versus SU; (ii) SGLT2i versus SU; and (iii) SGLT2i versus DPP4i; with time since baseline set as the timescale. Three sets of adjustments were used: (1) unadjusted; (2) partially adjusted for sex, age, IMD, time since type 2 diabetes diagnosis and region; (3) and adjusted for all pre‐specified covariates. Age, type 2 diabetes duration, HbA1c, BMI and eGFR at baseline were modelled continuously using 4‐knot restricted cubic splines. To obtain overall population estimates, ethnicity was added to the model as a covariate. To obtain ethnic‐specific estimates, an interaction term between ethnicity and treatment group was added to the model. A joint Wald interaction test assessed multiplicative interactions between ethnicity and treatment class for DPP4i versus SU, SGLT2i versus SU and DPP4i versus SGLT2i and MACE across ethnic groups. Given the higher power required to detect interactions at the multiplicative level, we discuss evidence of *p*‐interaction values close to 0.10.

### 2.5 Sensitivity analyses

Sensitivity analyses were conducted by repeating the primary analysis making the following adaptations one at a time. Firstly, complete case analysis was used to handle missing covariate data to assess the robustness of results to assumptions made about missingness in the main analysis. Secondly, we used a per‐protocol exposure definition, where follow‐up was truncated at the earliest of treatment switching, adding a new glucose‐lowering treatment, or after a gap in prescription records of 60 days or more to assess the impact of limiting the exposure to treatment periods. Thirdly, to address potential informative censoring in the per‐protocol sensitivity analysis due to treatment discontinuation or switching being related to the risk of outcome, we conducted another sensitivity analysis using a per‐protocol exposure definition in a complete case sample, where we applied inverse probability of censoring weights (IPCW). Follow‐up was split into 30‐day intervals; at each interval we estimated the cumulative probability of remaining uncensored. Stabilised weights were applied to weighted Cox models to estimate HRs.

## 3 RESULTS

### 3.1 Descriptive results

**FIGURE 2:**
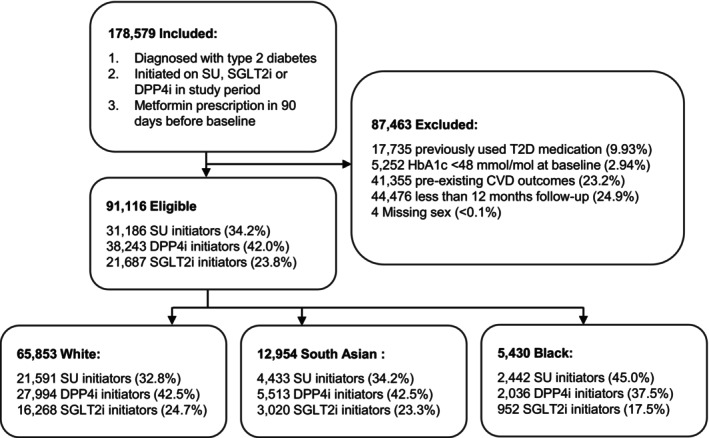
Flow diagram of study cohort selection. Adults with type 2 diabetes initiating sulfonylureas (SU), dipeptidyl peptidase‐4 inhibitors (DPP4i) or sodium‐glucose cotransporter‐2 inhibitors (SGLT2i) in addition to metformin were included and sequentially excluded based on eligibility criteria. The final study population (*N* = 91116) is shown by treatment group and by ethnic group. CVD, cardiovascular disease; HbA1c, glycated haemoglobin.

Of the 91 116 people who met the eligibility criteria (Figure 2, Table S3), 65 853 (72.3%) were White, 12 966 (14.2%) South Asian, 5430 (6.0%) Black, 1704 (1.9%) Other, 1001 (1.1%) Mixed and 1453 (1.6%) Not stated. Overall, there were 31 186 (34.2%) SU initiators, 38 243 (42.0%) DPP4i initiators and 21 687 (23.8%) SGLT2i initiators (Table 1). There were a lower proportion of SGLT2i initiators in the Black group (952, 17.5%) compared to the White (16 268, 24.7%) and South Asian (3020, 23.3%) groups. There were a higher proportion of SU initiators in the Black group (2442, 45.0%) than in the White (21 591, 32.8%) and South Asian (4433, 34.2%) groups (Table S4). There were 9154 (10.0%) individuals in the study population with missing covariate data, including 2709 (3.0%) with missing ethnicity data, five (<0.1%) missing IMD, 1455 (1.6%) missing HbA1c, 2985 (3.3%) missing BMI, 3546 (3.9%) missing eGFR and 11 (<0.1%) missing smoking status (Table 1).

**TABLE 1:** Baseline characteristics in the total study population by treatment group (*n* [col %] unless otherwise specified).

	SU initiators	DPP4i initiators	SGLT2i initiators	Total
Sample	31 186 (34.2)	38 243 (42.0)	21 687 (23.8)	91 116 (100.0)
Age, years, mean (SD)	57.4 (12.7)	60.1 (12.7)	54.8 (10.7)	57.9 (12.4)
Sex				
Male	18 302 (58.7)	21 626 (56.5)	12 420 (57.3)	52 348 (57.5)
Female	12 884 (41.3)	16 617 (43.5)	9267 (42.7)	38 768 (42.5)
Ethnicity				
White	21 591 (69.2)	27 994 (73.2)	16 268 (75.0)	65 853 (72.3)
South Asian	4433 (14.2)	5513 (14.4)	3020 (13.9)	12 966 (14.2)
Black	2442 (7.8)	2036 (5.3)	952 (4.4)	5430 (6.0)
Other	667 (2.1)	684 (1.8)	353 (1.6)	1704 (1.9)
Mixed	398 (1.3)	391 (1.0)	212 (1.0)	1001 (1.1)
Not stated	528 (1.7)	533 (1.4)	392 (1.8)	1453 (1.6)
Missing	1127 (3.6)	1092 (2.9)	490 (2.3)	2709 (3.0)
IMD level				
1 (least deprived)	4458 (14.3)	5844 (15.3)	3748 (17.3)	14 050 (15.4)
2	5351 (17.2)	6855 (17.9)	3894 (18.0)	16 100 (17.7)
3	6008 (19.3)	7295 (19.1)	4176 (19.3)	17 479 (19.2)
4	7562 (24.2)	8764 (22.9)	4754 (21.9)	21 080 (23.1)
5 (most deprived)	7806 (25.0)	9483 (24.8)	5113 (23.6)	22 402 (24.6)
Missing	1 (<0.01)	2 (<0.01)	2 (<0.01)	5 (<0.01)
Calendar year at baseline				
2015	6919 (22.2)	4546 (11.9)	1123 (5.2)	12 588 (13.8)
2016	5457 (17.5)	5685 (14.9)	1513 (7.0)	12 655 (13.9)
2017	4525 (14.5)	6197 (16.2)	2112 (9.7)	12 834 (14.1)
2018	3944 (12.7)	6534 (17.1)	2917 (13.5)	13 395 (14.7)
2019	3451 (11.1)	5805 (15.2)	3908 (18.0)	13 164 (14.5)
2020	2979 (9.6)	4490 (11.7)	3804 (17.7)	11 309 (12.4)
2021	3658 (11.7)	4624 (12.1)	5765 (26.6)	14 047 (15.4)
2022	253 (0.8)	362 (1.0)	509 (2.4)	1124 (1.2)
Region of England				
North East	1796 (5.8)	642 (1.7)	431 (2.0)	2869 (3.1)
North West	5129 (16.4)	7306 (19.1)	3856 (17.8)	16 291 (17.9)
Yorkshire and The Humber	940 (3.0)	1328 (3.5)	803 (3.7)	3071 (3.4)
East Midlands	807 (2.6)	913 (2.4)	283 (1.3)	2003 (2.2)
West Midlands	4526 (14.5)	7330 (19.2)	3512 (16.2)	15 368 (16.9)
East of England	1354 (4.3)	1479 (3.9)	764 (3.5)	3597 (3.9)
South West	8009 (25.7)	8188 (21.4)	3766 (17.4)	19 963 (21.9)
South Central	5362 (17.2)	6759 (17.7)	5680 (26.2)	17 801 (19.5)
London	3263 (10.5)	4298 (11.2)	2592 (12.0)	10 153 (11.1)
Type 2 diabetes duration, years (SD)	4.3 (4.3)	5.6 (4.5)	4.7 (4.0)	5.0 (4.4)
HbA1c level				
<53 mmol/mol (7%)	1049 (3.4)	1850 (4.8)	741 (3.4)	3640 (4.0)
53–74 mmol/mol (7–9%)	12 113 (38.8)	24 062 (62.9)	11 653 (53.7)	47 828 (52.5)
>75 mmol/mol (9%)	17 164 (55.0)	11 935 (31.2)	9094 (41.9)	38 193 (41.9)
Missing	860 (2.8)	396 (1.0)	199 (0.9)	1455 (1.6)
BMI category				
Underweight	108 (0.3)	71 (0.2)	8 (0.0)	187 (0.2)
Normal	3090 (9.9)	3069 (8.0)	625 (2.9)	6784 (7.4)
Overweight	9122 (29.3)	10 876 (28.4)	4088 (18.9)	24 086 (26.4)
Obese	17 152 (55.0)	23 355 (61.1)	16 567 (76.4)	57 074 (62.6)
Missing	1714 (5.5)	872 (2.3)	399 (1.8)	2985 (3.3)
eGFR				
≥60 mL/min/1.73 m^2^	27 656 (88.7)	34 091 (89.1)	20 772 (95.8)	82 519 (90.6)
<60 mL/min/1.73 m^2^	1721 (5.5)	3094 (8.1)	236 (1.1)	5051 (5.5)
Missing	1809 (5.8)	1058 (2.8)	679 (3.1)	3546 (3.9)
History of alcohol misuse				
No	28 690 (92.0)	35 645 (93.2)	20 093 (92.6)	84 428 (92.7)
Yes	2496 (8.0)	2598 (6.8)	1594 (7.4)	6688 (7.3)
Smoking status				
Never	7664 (24.6)	8880 (23.2)	5273 (24.3)	21 817 (23.9)
Current	8935 (28.7)	9912 (25.9)	5616 (25.9)	24 463 (26.8)
Former	14 582 (46.8)	19 447 (50.9)	10 796 (49.8)	44 825 (49.2)
Missing	5 (0.0)	4 (0.0)	2 (0.0)	11 (0.0)
Healthcare use				
≤10 consultation days	16 067 (51.5)	19 227 (50.3)	10 820 (49.9)	46 114 (50.6)
>10 consultation days	15 119 (48.5)	19 016 (49.7)	10 867 (50.1)	45 002 (49.4)
Comorbidities				
Atrial fibrillation	747 (2.4)	1151 (3.0)	441 (2.0)	2339 (2.6)
Peripheral artery disease	361 (1.2)	490 (1.3)	162 (0.7)	1013 (1.1)
Hypertension	14 811 (47.5)	21 276 (55.6)	11 171 (51.5)	47 258 (51.9)
Neuropathy	1151 (3.7)	1660 (4.3)	689 (3.2)	3500 (3.8)
Retinopathy	4100 (13.1)	6335 (16.6)	2845 (13.1)	13 280 (14.6)
All cancers	3237 (10.4)	4078 (10.7)	1442 (6.6)	8757 (9.6)
Chronic liver disease	302 (1.0)	320 (0.8)	152 (0.7)	774 (0.8)
Chronic respiratory diseases	1907 (6.1)	2385 (6.2)	945 (4.4)	5237 (5.7)
Rheumatoid arthritis	476 (1.5)	559 (1.5)	240 (1.1)	1275 (1.4)
Dementia	387 (1.2)	540 (1.4)	101 (0.5)	1028 (1.1)
Severe mental illness	869 (2.8)	898 (2.3)	497 (2.3)	2264 (2.5)
Common mental disorders	9344 (30.0)	11 154 (29.2)	7183 (33.1)	27 681 (30.4)
Medications				
ACE inhibitors	9937 (31.9)	14 295 (37.4)	7951 (36.7)	32 183 (35.3)
ARBs	3540 (11.4)	5492 (14.4)	2809 (13.0)	11 841 (13.0)
Calcium channel blockers	8021 (25.7)	11 207 (29.3)	5821 (26.8)	25 049 (27.5)
Diuretics	1448 (4.6)	2124 (5.6)	798 (3.7)	4370 (4.8)
Statins	19 486 (62.5)	27 767 (72.6)	14 823 (68.3)	62 076 (68.1)
Antiplatelets	2634 (8.4)	3670 (9.6)	1177 (5.4)	7481 (8.2)
Anticoagulants	916 (2.9)	1395 (3.6)	567 (2.6)	2878 (3.2)
Antipsychotics	686 (2.2)	694 (1.8)	414 (1.9)	1794 (2.0)

Abbreviations: ACE, angiotensin‐converting enzyme; ARBs, angiotensin II receptor blockers; BMI, body mass index; eGFR, estimated glomerular filtration rate; HbA1c, glycated haemoglobin; IMD, index of multiple deprivation; SD, standard deviation.

There cohort was 57.5% male and 42.5% female, which was broadly consistent across most groups, except Black DPP4i initiators and SGLT2i initiators, among whom there were more females than males. SGLT2i initiators were younger and had fewer pre‐existing comorbidities than other treatment groups. DPP4i initiators had a higher prevalence of microvascular conditions, hypertension and statin use than other treatment groups (Tables 1 and S4).

### 3.2 Crude incidence rates

The proportion of the study population that had ≥1 prescription for the same medication class after baseline was 90.8% in the overall population, 88.8% for SU initiators, 93.0% for DPP4i initiators and 89.7% for SGLT2i initiators. The median follow‐up time in the overall population for MACE was 2.9 years (interquartile range (IQR): 1.3–4.7); SGLT2i initiators had a shorter median follow‐up (2.0 years, IQR: 0.8–3.5) compared to DPP4i (3.1 years, IQR: 1.6–4.7) and SU initiators (3.5 years, IQR: 1.5–5.4). A total of 4274 MACE events were identified in the overall population, comprising 1859 events in SU initiators, 1942 events in DPP4i initiators and 473 events in SGLT2i initiators (Table S6A). There were very low event numbers for some individual cardiovascular outcomes in non‐White treatment groups, including myocardial infarction among Black SGLT2i initiators (*N* = 1) (Table S6B), stroke among Black SGLT2i initiators (*N* = 4) (Table S6C), heart failure among Black SGLT2i initiators (*N* = 2) (Table S6D) and cardiovascular death for South Asian SGLT2i initiators (*N* = 3) and Black SGLT2i initiators (*N* = 3) (Table S6E). The crude incidence rates for almost all cardiovascular outcomes were highest among SU initiators and lowest among SGLT2i initiators (Table S6).

### 3.3 Comparative effectiveness of medications by ethnicity

**FIGURE 3:**
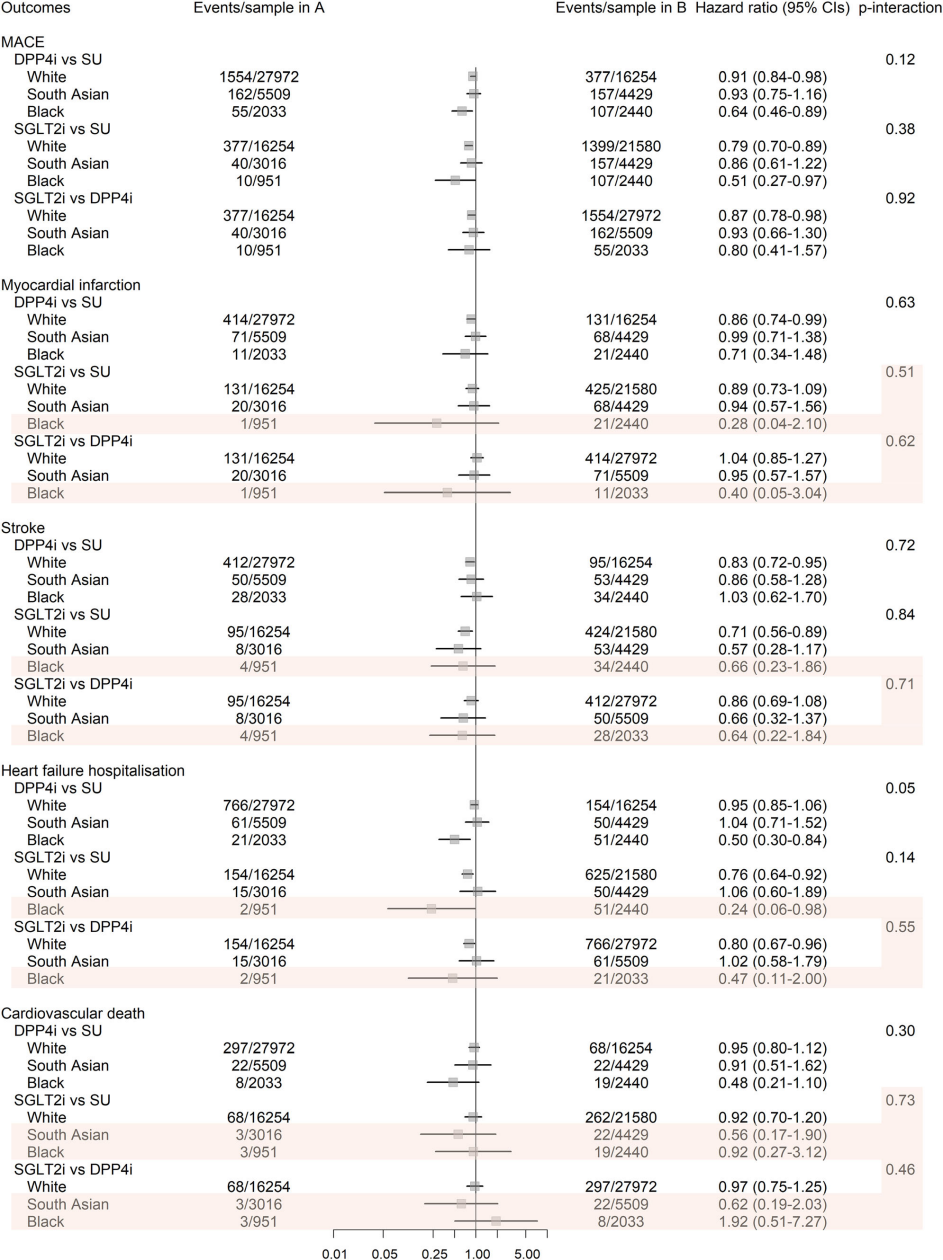
Forest plot of hazard ratios (HRs) and 95% confidence intervals (CIs) for adjusted analyses of interaction between ethnicity and treatment class on risk of cardiovascular outcomes. HRs compare the risk of outcomes for the comparator treatment (Treatment A) versus reference treatment (Treatment B), with HRs <1 favouring the comparator and HRs >1 favouring the reference. ‘Events/sample in A’ and ‘Events/sample in B’ correspond to the number of events and sample sizes in the comparator and reference groups, respectively. Analyses shaded in red have ≤5 events and should be interpreted with caution due to unstable estimates. DPP4i, dipeptidyl peptidase‐4 inhibitors; MACE, major adverse cardiovascular events; SGLT2i, sodium‐glucose cotransporter‐2 inhibitors; SU, sulfonylureas.

There was weak evidence of ethnic differences in the comparative effectiveness of DPP4i versus SU on MACE hazard (*p*‐interaction = 0.12), with DPP4i associated with a large reduction in MACE hazard in the Black group (HR: 0.64, 95% CI: 0.46–0.89), a modest reduction in the White group (HR: 0.91, 95% CI: 0.84–0.92) and no statistical evidence of an effect in the South Asian group (HR: 0.93, 95% CI: 0.75–1.16). There was no statistical evidence of ethnic differences in the comparative effectiveness of SGLT2i versus SU (*p*‐interaction = 0.38); however, SGLT2i were associated with a very large reduction in MACE hazard in the Black group (HR: 0.51, 95% CI: 0.27–0.97), a relatively large reduction in MACE hazard in the White group (HR: 0.79, 95% CI: 0.70–0.89) and no clear evidence in the South Asian group (HR: 0.86, 95% CI: 0.61–1.22). For SGLT2i compared to DPP4i, there was no statistical evidence of ethnic differences in the comparative effectiveness of treatment (*p*‐interaction = 0.92). Compared with DPP4i, there was weak evidence that SGLT2i were associated with a lower hazard of MACE in the White group (HR: 0.87, 95% CI: 0.78–0.98), but no clear evidence in the South Asian (HR: 0.93, 95% CI: 0.66–1.30) or Black (HR: 0.80, 95% CI: 0.41–1.57) groups (Figure 3, Table S7A).

For individual cardiovascular outcomes, there was evidence of ethnic differences for DPP4i versus SU on heart failure hospitalisation (*p*‐interaction = 0.05), with DPP4i associated with a large reduction in the hazard of heart failure hospitalisation in the Black group (HR: 0.50, 95% CI: 0.30–0.84), a modest reduction in the White group (HR: 0.95, 95% CI: 0.85–1.06) and no clear effect in the South Asian group (HR: 1.04, 95% CI: 0.71–1.52). There was no evidence of ethnic differences in the association between any other treatment comparisons and cardiovascular outcomes. Some treatment comparisons for individual cardiovascular outcomes were underpowered and unstable due to sparse events, specifically the Black group for all outcomes when comparing SGLT2i versus SU and SGLT2i versus DPP4i, the Black group for cardiovascular death when comparing DPP4i versus SU and the South Asian group for cardiovascular death when comparing SGLT2i versus SU and SGLT2i versus DPP4i (Figure 3; Table S7B–E).

### 3.4 Sensitivity analyses

In the complete case analysis, the primary estimates were consistent with the main analysis (Table S8). Findings were broadly consistent when a per‐protocol exposure definition was used, where follow‐up was truncated after a gap in prescription records of 60 days or more; treatment switching or adding, findings were consistent (Table S9). Findings remained broadly consistent when a per‐protocol exposure definition was used, and IPCW was applied to account for potential informative censoring (Table S10).

## 4 DISCUSSION

### 4.1 Overall findings

In this retrospective cohort study of 91 116 adults with type 2 diabetes initiating second‐line treatment with SU, DPP4i or SGLT2i and metformin, there was weak evidence of ethnic differences in the comparative effectiveness of DPP4i versus SU treatments on MACE, with a stronger effect observed for DPP4i in the Black group than in other ethnic groups. There was evidence of ethnic differences in DPP4i versus SU on heart failure hospitalisation, for which a stronger effect was observed for DPP4i in the Black group.

### 4.2 Findings in context

Few studies have directly compared the cardiovascular effect of second‐line type 2 diabetes medications across ethnic groups in real‐world settings. Several cardiovascular outcome trials of SGLT2i versus placebo have reported effects in ethnic subgroups, including the DECLARE‐TIMI 58 (Dapagliflozin Effect on CardiovascuLAR Events) trial, DAPA‐HF (Dapagliflozin and Prevention of Adverse Outcomes in Heart Failure) trial, EMPA‐REG OUTCOME (BI 10773 [Empagliflozin] Cardiovascular Outcome Event Trial in Type 2 Diabetes Mellitus Patients) trial and CANVAS (CANagliflozin cardioVascular Assessment Study) trial.^15^, ^16^, ^18^, ^21^ The DAPA‐HF trial observed stronger protective effects for heart failure hospitalisation in Black (HR 0.62, 95% CI 0.37–1.04) and South Asian participants (HR 0.64, 95% CI 0.48–0.86) compared with White participants (HR 0.78, 95% CI 0.66–0.91),^18^ whereas the other trials found no clear ethnic differences for MACE.^15^, ^16^, ^21^ The Cardiovascular Events Associated With SGLT‐2 Inhibitors Versus Other Glucose‐Lowering Drugs study, a large international cohort spanning Singapore, Japan, Korea, Israel, Canada and Australia, found SGLT2i to be more cardioprotective than other glucose‐lowering medications, with stronger effects observed for heart failure in non‐Asian settings but no clear regional differences for other cardiovascular outcomes.^28^ However, comparisons between the findings from these studies and the current study are limited due to important differences in study designs. Firstly, these studies did not directly compare specific second‐line medication classes. Secondly, most included participants from multiple countries, whereas our study was based on a UK population. It remains unclear to what extent cross‐country comparisons can provide meaningful insights into ethnic‐specific treatment effects, as ethnicity is context specific, shaped by local demographic, social and clinical factors.

The stronger effect of DPP4i compared with SU on heart failure hospitalisation in the Black group may reflect a combination of factors, from biological mechanisms to broader social determinants of health. Although ethnic differences in adherence to glucose‐lowering medications have been linked to treatment outcome disparities,^29^ this study compared the effectiveness of medications within each ethnic group, so ethnic differences in adherence may be less relevant. Evidence has indicated that Black people have a higher underlying risk of heart failure,^10^, ^11^, ^12^, ^13^, ^14^ possibly contributing to the more pronounced differences between medications observed for heart failure hospitalisation in the Black group.

### 4.3 Strengths

Our study included a large study population that was broadly representative of the UK population, in terms of ethnicity and other demographic characteristics.^30^, ^31^ We assessed the effectiveness of the medications when used in real‐world settings. The study period was limited to a time when UK therapeutic guidelines did not provide strong recommendations on which medication should be prescribed to whom, reducing confounding by indication. We only included new initiators of second‐line treatment to avoid prevalent user bias, which can occur when comparing new initiators, who can experience early medication risks, with prevalent initiators, who have ‘survived’ the early risks.^32^ We used an active comparator where all included individuals were prescribed a second‐line medication, which increased the likelihood of comparing individuals with similar disease severity. Ethnicity data collected in UK clinical settings are usually self‐reported using standardised categories, which are recommended for consistency and validity.^33^, ^34^, ^35^ CPRD‐HES ethnicity coverage exceeds 80%, and fewer than 10% of people have discordant ethnicity records at the most granular level, although discordance rates are higher in minority ethnic groups.^36^

### 4.4 Limitations

Several limitations should be considered when interpreting these findings. Despite adjusting for measured confounders, there is likely to be some unmeasured confounding, such as dietary habits or exercise. The use of SU as second‐line therapy may be, in part, cost‐driven, which may introduce confounding when comparing with other therapies. However, we mitigated this potential confounding by adjusting all models for IMD, which accounted for socioeconomic differences between people in different treatment groups. Exposure was defined using prescription records, which do not guarantee that the medication is taken as directed, resulting in potential exposure misclassification. Although we started with a large national sample, a smaller proportion of people were initiated on a SGLT2i, which was new during the study period, and there were smaller numbers of non‐White people in our study population. This resulted in very sparse event numbers for some analyses, resulting in unstable estimates. We used broad ethnic categories to increase the sample sizes, which may have obscured treatment effect differences in more specific ethnic subgroups. However, these standardised categories align with those commonly used in research and monitoring in the United Kingdom, enhancing comparability.^37^, ^38^, ^39^ We did not examine ethnic differences in the cardiovascular effects of other recommended second‐line classes, such as glucagon‐like peptide‐1 receptor agonists (GLP‐1RA), which have proven cardiovascular benefits, because their low uptake during our study period resulted in an insufficient sample size. We were unable to capture important explanatory variables for ethnic disparities, such as genetic variation.

## 5 CONCLUSIONS

We found weak evidence suggesting ethnic differences in the effect of DPP4i compared to SU on MACE, with a stronger effect of DPP4i observed in the Black group. This may be related to evidence of a stronger protective effect of DPP4i compared to SU for heart failure hospitalisation in the Black group. Further research using data sources including more non‐White people is needed to confirm these findings.

## AUTHOR CONTRIBUTIONS

MH, RM and EW conceived the study. MH, RM, EW and CTR designed the methodology. ASVS and CW‐G assisted with covariate and outcome definitions. MH undertook the analyses, with support and supervision from RM, EW and CTR. PB and MB assisted with software. MH prepared the first draft of the manuscript. All contributing authors reviewed and edited the manuscript.

## FUNDING INFORMATION

This work was supported by the Medical Research Council (MRC) (MR/N013638/1), by Barts Charity (MGU0504) and the Wellcome Trust (225 868/Z/22/Z).

## CONFLICT OF INTEREST STATEMENT

Marleen Bokern was funded by a GlaxoSmithKline PhD studentship during the conduct of this study. All other co‐authors disclose no conflicts of interest.

## Supporting information


**Data S1.** Supporting Information.

## Data Availability

Summary data are available upon reasonable request. Access to underlying data need to be requested and approved by the study team of CPRD.
