# Supplementary material for: Ethnic differences in the comparative effectiveness of second‐line type 2 diabetes medications in preventing cardiovascular disease
Source: Diabetes Obes Metab. 2025 Nov 12;28(2):1013–23. doi: 10.1111/dom.70273 (PMC12803632; doi:10.1111/dom.70273)
Supplement: Supplementary file 1 — Data S1. Supporting Information. [file DOM-28-1013-s001.docx]

**Supplementary tables and figures**

[Figure S1: Directed Acyclic Graph (DAG) showing causal assumptions about the relationship between measured covariates, exposure and outcome. 2](#_Toc213425540)

[Table S1: Inclusion and exclusion criteria and how they were defined 3](#_Toc213425541)

[Table S2: Cardiovascular outcomes and how they were defined 5](#_Toc213425542)

[Table S3: Baseline covariates and how they were defined 6](#_Toc213425543)

[Table S4: Baseline characteristics by treatment group and ethnic group (n (col %) unless otherwise specified) 8](#_Toc213425544)

[Table S5: Baseline characteristics in missing and no missing. All statistics reported as number (% of column total), unless stated otherwise. 12](#_Toc213425545)

[Table S6: Crude incidence rates by treatment group and ethnic group for each outcome 15](#_Toc213425546)

[Table S7: Hazard ratios and 95% confidence intervals for the interaction between ethnicity and medication class on risk of cardiovascular outcomes 19](#_Toc213425547)

[Table S8. Sensitivity analysis: Hazard ratios and 95% confidence intervals for interaction between ethnicity and medication class on risk of MACE using complete case sample 22](#_Toc213425548)

[Table S9. Sensitivity analysis: interaction between treatment class and ethnicity on risk of MACE using per-protocol exposure definition where follow-up was truncated after treatment stopping, switching or adding 23](#_Toc213425549)

[Table S10. Sensitivity analysis: interaction between treatment class and ethnicity on risk of MACE using per-protocol exposure definition where follow-up was truncated after treatment stopping, switching or adding, and inverse probability of censoring weights (IPCW) applied to account for informative censoring 24](#_Toc213425550)

[Table S11: Adjusted Cox failure curves for MACE by treatment group for each ethnic group 25](#_Toc213425551)

[References 29](#_Toc213425552)

## Figure S1: Directed Acyclic Graph (DAG) showing causal assumptions about the relationship between measured covariates, exposure and outcome.


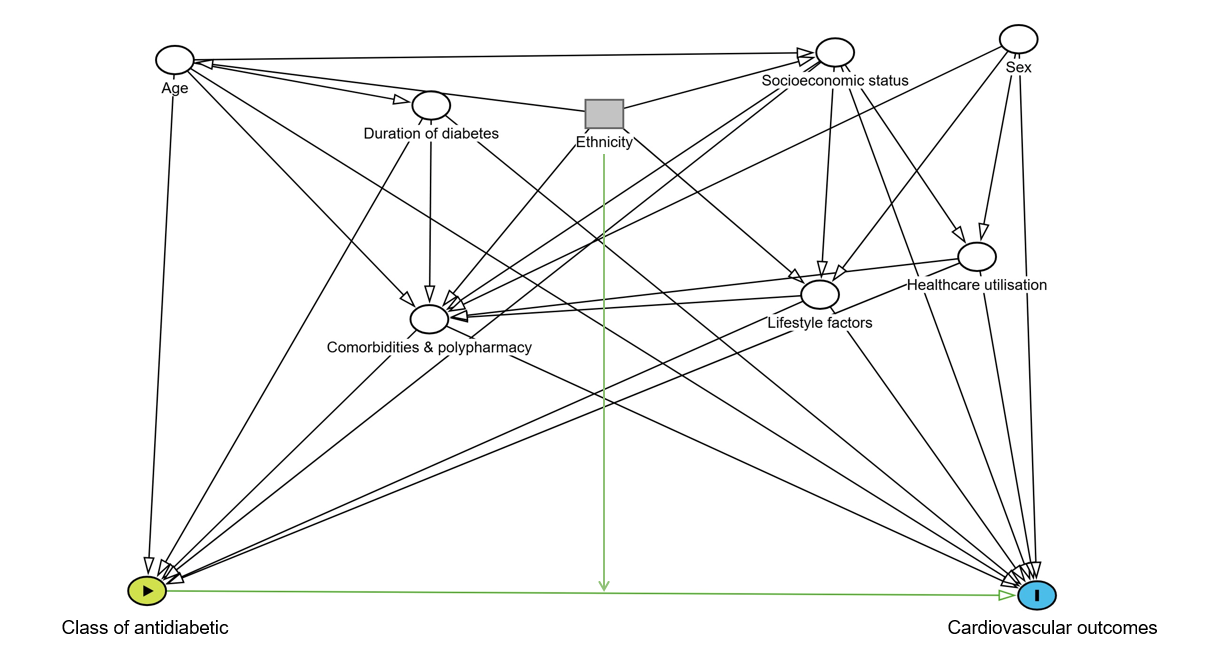


## Table S1: Inclusion and exclusion criteria and how they were defined

| **Inclusion criteria** | **Data source** | **Definition** |
| --- | --- | --- |
| Type 2 diabetes diagnosis | CPRD | Type 2 diabetes diagnostic code at 18 years or over |
| Initiation on SU, DPP4i or SGLT2i | CPRD | First prescription of either SU, DPP4i or SGLT2i during the study period (1st January 2015 and 1st February 2022), excluding people who initiated on >1 medication on the same day. |
| Metformin prescription at baseline | CPRD | Record of prescription of metformin in 90 days before first prescription of SU, DPP4i or SGLT2i |

| **Exclusion criteria** | **Data source** | **Definition** |
| --- | --- | --- |
| Antidiabetic medication before baseline | CPRD | Record of any antidiabetic medication (except metformin but including exposure medications) before baseline. |
| HbA1c <48 mmol/mol (6.5%) at baseline | CPRD | HbA1c (mmol/mol) was determined using values from within two years before baseline, based on the following priority: 1. Median of recordings from within 0–3 months before baseline; 2. If unavailable, median of recordings from 3–6 months before baseline; 3. If still unavailable, median of recordings from 6–12 months before baseline; 4. Finally, median of recordings from 1–2 years before baseline. |
| Pre-existing cardiovascular conditions before baseline | CPRD  HES | Record of pre-existing myocardial infarction, stroke, heart failure, ischaemic heart disease before baseline, defined by incident and prevalent codes in CPRD and any events in HES. |
| Less than 12 months follow up before baseline | CPRD | Less than 12 months continuous follow-up before baseline |
| Missing sex data | CPRD | Missing sex data |

Abbreviations: CPRD, Clinical Practice Research Datalink; SU, sulfonylurea; DPP4i, dipeptidyl peptidase 4 inhibitor; SGLT2i, and sodium-glucose cotransporter 2 inhibitor; HES, Hospital Episode Statistics.

## Table S2: Cardiovascular outcomes and how they were defined

| **Outcome** | **Data source** | **Definition** |
| --- | --- | --- |
| Myocardial infarction | CPRD  HES | Earliest event of those detected in primary care records using incident codes indicating myocardial infarction and in HES APC using ICD10 codes: I21*, I22* and I23* |
| Stroke | CPRD  HES | Earliest event of those detected in primary care records using incident codes indicating haemorrhagic and ischaemic (but non-traumatic) stroke and in HES Admitted Patient Care using ICD10 codes: I60*, I61*, I62.0 I62.1, I62.9, I63*, I64 |
| Cardiovascular death | ONS | Detected in ONS death records where ICD10 codes beginning with I* (indicating any disease of the circulatory system) were recorded as the primary cause of death. |
| Heart failure hospitalisation | CPRD  ONS | Earliest event detected in HES APC using ICD10 I11.0, I13.0, I13.2, I50, I50.0, I50.9 |

Abbreviations: ONS, Office of National Statistics.

## Table S3: Baseline covariates and how they were defined

| **Covariate** | **Data source** | **Definition** |
| --- | --- | --- |
| Age | CPRD | Month and year of birth from CPRD datasets were combined and middle of the month was used as the date. |
| Sex | CPRD | CPRD gender variable, which is typically self-reported and assumed to refer to biological sex(1). |
| Ethnicity | CPRD | Ethnicity is often recorded multiple times in UK electronic health records, with a small proportion of individuals with discordant recordings (<10%)(2). A previously developed algorithm was used to determine each individuals ethnic group based on the frequency and recency of recordings(3). Ethnicity was categorized as White, South Asian, Black, Other, Mixed or Not Stated |
| IMD | CPRD | CPRD-linked IMD is derived by mapping patient postcode to small-area deprivation data. The GP postcode was used when the patient postcode was unavailable. IMD was categorized in quintiles from 1 (least deprived) to 5 (most deprived) |
| Region of England | CPRD | Region of England where the GP practice is based: North East, North West, Yorkshire And The Humber, East Midlands, West Midlands, East of England, South West, South Central, London |
| Calendar year at baseline | CPRD | Calendar year at baseline was used to account for changes in prescribing patterns and clinical practice throughout the study period. |
| Duration of type 2 diabetes | CPRD | Type 2 diabetes diagnosis date defined as the earliest diagnostic code in primary care records. People without type 2 diabetes diagnosis before baseline were excluded (**Table S2**). Duration of type 2 diabetes calculated as years from type 2 diabetes diagnosis to baseline. |
| HbA1c level | CPRD | HbA1c (mmol/mol) was determined using values from within two years before baseline, based on the following priority: 1. Median of recordings from within 0–3 months before baseline; 2. If unavailable, median of recordings from 3–6 months before baseline; 3. If still unavailable, median of recordings from 6–12 months before baseline; 4. Finally, median of recordings from 1–2 years before baseline. |
| BMI | CPRD | BMI (kg/m²) was either calculated from weight and height measurements, or from a GP-recorded BMI measurement was used, with calculated BMI prioritised. The BMI measurement nearest to baseline from within 5 years before baseline was used, based on a validated algorithm(4). BMI values were categorised separately for South Asian and Black individuals and all other ethnic groups, in accordance with NHS ethnic-specific cut-offs (5). South Asian and Black individuals were classified as underweight at <18.5 kg/m2, healthy at 18.5-22.9 kg/m2, overweight at 23-27.4kg/m2, and obese at ≥27.5 kg/m2. Individuals from all other ethnic groups were categorised as underweight at <18.5 kg/m^2^, healthy at 18.5-24.9 kg/m^2^, overweight at 25-29.9 kg/m^2^, and obese ≥30 kg/m^2^. |
| eGFR | CPRD | Baseline eGFR (mL/min/1.73 m²) was estimated using the most recently serum creatine level (µmol/L) measurement for each person in the 24 months before baseline. eGFR was calculated from serum creatine level using the Chronic Kidney Disease Epidemiology Consortium (CKD-EPI) equation(6). We did not use an ethnic-specific eGFR calculation because this is no longer considered valid or accurate(7, 8). |
| History of alcohol misuse | CPRD | One or more clinical code indicating excessive alcohol consumption, addiction, alcohol-related health condition, alcohol-related accident or incident, referral to alcohol services or a record of being prescribed medication for alcohol addiction, ever before baseline. |
| Smoking status | CPRD | Smoking status was determined using an algorithm that assessed all recordings before baseline to categorise an individual’s consumption status as never, current and former. |
| Rate of primary care consultations | CPRD | Unique consultation days were defined as in-person appointments, virtual appointments and telephone appointments on different days. Rate of consultations was categorised as ≤10 or >10 consultation days, based on an average of 12 consultation days in the study population and external data showing an average of ~10 primary care attendances per year among type 2 diabetes patients (11) |
| Medications | CPRD | Binary variables created for each medication, defined as the presence of one or more prescription records in the year before baseline. |
| Pre-existing comorbidities | CPRD | Binary variables created for each co-morbidity, defined as the presence of one or more relevant clinical codes before baseline. |

Abbreviations: IMD, index of multiple deprivation; HbA1c, glycated haemoglobin; BMI, body mass index.

## Table S4: Baseline characteristics by treatment group and ethnic group (n (col %) unless otherwise specified)

|  | **White SU** | **White DPP4i** | **White SGLT2i** | **South Asian SU** | **South Asian DPP4i** | **South Asian SGLT2i** | **Black SU** | **Black DPP4i** | **Black SGLT2i** |
| --- | --- | --- | --- | --- | --- | --- | --- | --- | --- |
| **Sample** | 21591 (32.8) | 27994 (42.5) | 16268 (24.7) | 4433 (34.2) | 5513 (42.5) | 3020 (23.3) | 2442 (45.0) | 2036 (37.5) | 952 (17.5) |
| **Age, mean years (SD)** | 59.2 (12.5) | 61.8 (12.3) | 55.9 (10.6) | 51.3 (11.8) | 52.8 (11.8) | 49.8 (10.4) | 53.7 (11.2) | 55.9 (12.0) | 52.9 (9.4) |
| **Sex** |  |  |  |  |  |  |  |  |  |
| Male | 12635 (58.5) | 15904 (56.8) | 9367 (57.6) | 2641 (59.6) | 3118 (56.6) | 1728 (57.2) | 1392 (57.0) | 1016 (49.9) | 459 (48.2) |
| Female | 8956 (41.5) | 12090 (43.2) | 6901 (42.4) | 1792 (40.4) | 2395 (43.4) | 1292 (42.8) | 1050 (43.0) | 1020 (50.1) | 493 (51.8) |
| **IMD level** |  |  |  |  |  |  |  |  |  |
| 1 (least deprived) | 3605 (16.7) | 4868 (17.4) | 3135 (19.3) | 345 (7.8) | 434 (7.9) | 275 (9.1) | 68 (2.8) | 60 (2.9) | 51 (5.4) |
| 2 | 4217 (19.5) | 5646 (20.2) | 3167 (19.5) | 552 (12.5) | 652 (11.8) | 406 (13.4) | 142 (5.8) | 124 (6.1) | 62 (6.5) |
| 3 | 4229 (19.6) | 5418 (19.4) | 3123 (19.2) | 915 (20.6) | 1043 (18.9) | 603 (20.0) | 338 (13.8) | 285 (14.0) | 161 (16.9) |
| 4 | 4560 (21.1) | 5759 (20.6) | 3265 (20.1) | 1415 (31.9) | 1653 (30.0) | 867 (28.7) | 903 (37.0) | 719 (35.3) | 313 (32.9) |
| 5 (most deprived) | 4979 (23.1) | 6302 (22.5) | 3578 (22.0) | 1206 (27.2) | 1730 (31.4) | 868 (28.7) | 991 (40.6) | 848 (41.7) | 365 (38.3) |
| Missing | 1 (0.0) | 1 (0.0) | 0 (0.0) | 0 (0.0) | 1 (0.0) | 1 (0.0) | . (.) | . (.) | . (.) |
| **Calendar year at baseline** |  |  |  |  |  |  |  |  |  |
| 2015 | 2353 (7.8) | 845 (2.2) | 564 (2.7) | 51 (0.9) | 20 (0.3) | 10 (0.3) | 14 (0.5) | 9 (0.4) | 0 (0.0) |
| 2016 | 6010 (20.0) | 8842 (22.5) | 4210 (20.2) | 582 (10.8) | 880 (13.0) | 556 (15.3) | 110 (3.8) | 133 (5.5) | 65 (5.9) |
| 2017 | 1086 (3.6) | 1648 (4.2) | 893 (4.3) | 50 (0.9) | 78 (1.2) | 55 (1.5) | 9 (0.3) | 21 (0.9) | 10 (0.9) |
| 2018 | 932 (3.1) | 1026 (2.6) | 325 (1.6) | 109 (2.0) | 108 (1.6) | 33 (0.9) | 30 (1.0) | 17 (0.7) | 8 (0.7) |
| 2019 | 4673 (15.5) | 7658 (19.5) | 3396 (16.3) | 853 (15.8) | 1482 (21.9) | 684 (18.9) | 207 (7.1) | 224 (9.3) | 118 (10.7) |
| 2020 | 1468 (4.9) | 1604 (4.1) | 812 (3.9) | 190 (3.5) | 196 (2.9) | 93 (2.6) | 33 (1.1) | 39 (1.6) | 17 (1.5) |
| 2021 | 3999 (13.3) | 4747 (12.1) | 2098 (10.1) | 2854 (52.8) | 3114 (46.0) | 1472 (40.6) | 2197 (75.6) | 1718 (71.3) | 689 (62.4) |
| 2022 | 5591 (18.6) | 7711 (19.7) | 5754 (27.6) | 570 (10.5) | 668 (9.9) | 534 (14.7) | 196 (6.7) | 119 (4.9) | 130 (11.8) |
| **Region of England** |  |  |  |  |  |  | 112 (3.9) | 129 (5.4) | 68 (6.2) |
| North East | 1670 (7.7) | 601 (2.1) | 412 (2.5) | 44 (1.0) | 13 (0.2) | 8 (0.3) | 11 (0.5) | 9 (0.4) | 0 (0.0) |
| North West | 4214 (19.5) | 6053 (21.6) | 3158 (19.4) | 453 (10.2) | 703 (12.8) | 454 (15.0) | 87 (3.6) | 117 (5.7) | 53 (5.6) |
| Yorkshire | 795 (3.7) | 1143 (4.1) | 672 (4.1) | 40 (0.9) | 63 (1.1) | 47 (1.6) | 7 (0.3) | 21 (1.0) | 10 (1.1) |
| East Midlands | 637 (3.0) | 749 (2.7) | 230 (1.4) | 77 (1.7) | 75 (1.4) | 25 (0.8) | 25 (1.0) | 18 (0.9) | 7 (0.7) |
| West Midlands | 3349 (15.5) | 5541 (19.8) | 2694 (16.6) | 704 (15.9) | 1200 (21.8) | 552 (18.3) | 172 (7.0) | 186 (9.1) | 93 (9.8) |
| East of England | 1055 (4.9) | 1199 (4.3) | 628 (3.9) | 142 (3.2) | 143 (2.6) | 73 (2.4) | 30 (1.2) | 34 (1.7) | 13 (1.4) |
| South West | 3001 (13.9) | 3547 (12.7) | 1654 (10.2) | 2371 (53.5) | 2571 (46.6) | 1215 (40.2) | 1855 (76.0) | 1434 (70.4) | 596 (62.6) |
| South Central | 4148 (19.2) | 5501 (19.7) | 4637 (28.5) | 479 (10.8) | 552 (10.0) | 481 (15.9) | 157 (6.4) | 103 (5.1) | 120 (12.6) |
| London | 2722 (12.6) | 3660 (13.1) | 2183 (13.4) | 123 (2.8) | 193 (3.5) | 165 (5.5) | 98 (4.0) | 114 (5.6) | 60 (6.3) |
| **Type 2 diabetes duration, years (SD)** | 4.3 (4.3) | 5.7 (4.5) | 4.7 (4.0) | 4.6 (4.3) | 5.5 (4.3) | 5.0 (4.0) | 4.1 (4.3) | 5.5 (4.5) | 5.1 (4.1) |
| **HbA1c level** |  |  |  |  |  |  |  |  |  |
| <53 mmol/mol (7%) | 725 (3.4) | 1387 (5.0) | 574 (3.5) | 142 (3.2) | 238 (4.3) | 94 (3.1) | 75 (3.1) | 101 (5.0) | 29 (3.0) |
| 53-74 mmol/mol (7-9%) | 8361 (38.7) | 17703 (63.2) | 8665 (53.3) | 1979 (44.6) | 3612 (65.5) | 1762 (58.3) | 789 (32.3) | 1115 (54.8) | 470 (49.4) |
| >75 mmol/mol (9%) | 11904 (55.1) | 8611 (30.8) | 6877 (42.3) | 2224 (50.2) | 1615 (29.3) | 1140 (37.7) | 1500 (61.4) | 798 (39.2) | 447 (47.0) |
| Missing | 601 (2.8) | 293 (1.0) | 152 (0.9) | 88 (2.0) | 48 (0.9) | 24 (0.8) | 78 (3.2) | 22 (1.1) | 6 (0.6) |
| **BMI Category** |  |  |  |  |  |  |  |  |  |
| Underweight | 81 (0.4) | 48 (0.2) | 3 (0.0) | 15 (0.3) | 8 (0.1) | 4 (0.1) | 4 (0.2) | 8 (0.4) | 0 (0.0) |
| Normal | 2120 (9.8) | 2226 (8.0) | 421 (2.6) | 423 (9.5) | 390 (7.1) | 101 (3.3) | 136 (5.6) | 87 (4.3) | 17 (1.8) |
| Overweight | 5987 (27.7) | 7658 (27.4) | 2894 (17.8) | 1611 (36.3) | 1918 (34.8) | 748 (24.8) | 628 (25.7) | 437 (21.5) | 121 (12.7) |
| Obese | 12270 (56.8) | 17517 (62.6) | 12678 (77.9) | 2161 (48.7) | 3047 (55.3) | 2107 (69.8) | 1556 (63.7) | 1442 (70.8) | 799 (83.9) |
| Missing | 1133 (5.2) | 545 (1.9) | 272 (1.7) | 223 (5.0) | 150 (2.7) | 60 (2.0) | 118 (4.8) | 62 (3.0) | 15 (1.6) |
| **eGFR** |  |  |  |  |  |  |  |  |  |
| ≥60 mL/min/1.73 m² | 18939 (87.7) | 24659 (88.1) | 15570 (95.7) | 4133 (93.2) | 5215 (94.6) | 2920 (96.7) | 2206 (90.3) | 1848 (90.8) | 899 (94.4) |
| <60 mL/min/1.73 m² | 1342 (6.2) | 2519 (9.0) | 164 (1.0) | 105 (2.4) | 197 (3.6) | 28 (0.9) | 119 (4.9) | 156 (7.7) | 32 (3.4) |
| Missing | 1310 (6.1) | 816 (2.9) | 534 (3.3) | 195 (4.4) | 101 (1.8) | 72 (2.4) | 117 (4.8) | 32 (1.6) | 21 (2.2) |
| **History of alcohol abuse** |  |  |  |  |  |  |  |  |  |
| Yes | 19680 (91.1) | 26001 (92.9) | 14982 (92.1) | 4190 (94.5) | 5182 (94.0) | 2861 (94.7) | 2275 (93.2) | 1900 (93.3) | 885 (93.0) |
| No | 1911 (8.9) | 1993 (7.1) | 1286 (7.9) | 243 (5.5) | 331 (6.0) | 159 (5.3) | 167 (6.8) | 136 (6.7) | 67 (7.0) |
| **Smoking status** |  |  |  |  |  |  |  |  |  |
| Never | 4394 (20.4) | 5618 (20.1) | 3458 (21.3) | 1647 (37.2) | 1868 (33.9) | 1086 (36.0) | 856 (35.1) | 632 (31.0) | 281 (29.5) |
| Current | 6505 (30.1) | 7483 (26.7) | 4398 (27.0) | 1064 (24.0) | 1301 (23.6) | 652 (21.6) | 561 (23.0) | 471 (23.1) | 225 (23.6) |
| Former | 10690 (49.5) | 14893 (53.2) | 8412 (51.7) | 1720 (38.8) | 2344 (42.5) | 1282 (42.5) | 1025 (42.0) | 933 (45.8) | 446 (46.8) |
| Missing | 2 (<0.01) | 0 (<0.01) | 0 (<0.01) | 2(<0.01) | 0 (<0.01) | 0 (<0.01) | 0 (<0.01) | 0 (<0.01) | 0 (<0.01) |
| **Healthcare use** |  |  |  |  |  |  |  |  |  |
| ≤10 consultation days | 10761 (49.8) | 13972 (49.9) | 8101 (49.8) | 2447 (55.2) | 2784 (50.5) | 1470 (48.7) | 1350 (55.3) | 1020 (50.1) | 489 (51.4) |
| >10 consultation days | 10830 (50.2) | 14022 (50.1) | 8167 (50.2) | 1986 (44.8) | 2729 (49.5) | 1550 (51.3) | 1092 (44.7) | 1016 (49.9) | 463 (48.6) |
| **Pre-existing comorbidities** |  |  |  |  |  |  |  |  |  |
| Atrial fibrillation | 639 (3.0) | 1007 (3.6) | 399 (2.5) | 14 (0.3) | 43 (0.8) | 9 (0.3) | 20 (0.8) | 15 (0.7) | 6 (0.6) |
| Peripheral artery disease | 315 (1.5) | 432 (1.5) | 146 (0.9) | 12 (0.3) | 14 (0.3) | 5 (0.2) | 8 (0.3) | 7 (0.3) | 3 (0.3) |
| Hypertension | 10674 (49.4) | 16199 (57.9) | 8699 (53.5) | 1731 (39.0) | 2448 (44.4) | 1271 (42.1) | 1226 (50.2) | 1211 (59.5) | 544 (57.1) |
| Neuropathy | 880 (4.1) | 1318 (4.7) | 561 (3.4) | 110 (2.5) | 183 (3.3) | 63 (2.1) | 64 (2.6) | 61 (3.0) | 22 (2.3) |
| Retinopathy | 2911 (13.5) | 4748 (17.0) | 2103 (12.9) | 581 (13.1) | 781 (14.2) | 400 (13.2) | 272 (11.1) | 336 (16.5) | 136 (14.3) |
| All cancers | 2633 (12.2) | 3530 (12.6) | 1241 (7.6) | 163 (3.7) | 183 (3.3) | 68 (2.3) | 140 (5.7) | 123 (6.0) | 40 (4.2) |
| Chronic liver disease | 231 (1.1) | 224 (0.8) | 112 (0.7) | 19 (0.4) | 34 (0.6) | 19 (0.6) | 23 (0.9) | 21 (1.0) | 7 (0.7) |
| Chronic respiratory diseases | 1624 (7.5) | 2079 (7.4) | 847 (5.2) | 94 (2.1) | 126 (2.3) | 45 (1.5) | 51 (2.1) | 53 (2.6) | 9 (0.9) |
| Rheumatoid arthritis | 379 (1.8) | 442 (1.6) | 201 (1.2) | 43 (1.0) | 67 (1.2) | 24 (0.8) | 23 (0.9) | 16 (0.8) | 7 (0.7) |
| Dementia | 277 (1.3) | 402 (1.4) | 83 (0.5) | 39 (0.9) | 47 (0.9) | 12 (0.4) | 29 (1.2) | 35 (1.7) | 5 (0.5) |
| Severe mental illness | 585 (2.7) | 648 (2.3) | 366 (2.2) | 118 (2.7) | 114 (2.1) | 71 (2.4) | 97 (4.0) | 75 (3.7) | 41 (4.3) |
| Common mental disorders | 7502 (34.7) | 9190 (32.8) | 6120 (37.6) | 737 (16.6) | 943 (17.1) | 525 (17.4) | 386 (15.8) | 349 (17.1) | 161 (16.9) |
| **Medications in the year before index** |  |  |  |  |  |  |  |  |  |
| ACE inhibitors | 7277 (33.7) | 11020 (39.4) | 6293 (38.7) | 1251 (28.2) | 1716 (31.1) | 919 (30.4) | 625 (25.6) | 602 (29.6) | 260 (27.3) |
| Angiotensin II receptor blockers | 2469 (11.4) | 4067 (14.5) | 2086 (12.8) | 498 (11.2) | 766 (13.9) | 404 (13.4) | 284 (11.6) | 305 (15.0) | 134 (14.1) |
| Calcium channel blockers | 5398 (25.0) | 8154 (29.1) | 4371 (26.9) | 968 (21.8) | 1347 (24.4) | 660 (21.9) | 966 (39.6) | 930 (45.7) | 421 (44.2) |
| Diuretics | 1188 (5.5) | 1784 (6.4) | 680 (4.2) | 68 (1.5) | 122 (2.2) | 45 (1.5) | 68 (2.8) | 77 (3.8) | 35 (3.7) |
| Statins | 13580 (62.9) | 20491 (73.2) | 11204 (68.9) | 2959 (66.7) | 4120 (74.7) | 2111 (69.9) | 1352 (55.4) | 1312 (64.4) | 605 (63.6) |
| Antiplatelets | 1944 (9.0) | 2911 (10.4) | 934 (5.7) | 306 (6.9) | 389 (7.1) | 127 (4.2) | 144 (5.9) | 127 (6.2) | 38 (4.0) |
| Anticoagulants | 788 (3.6) | 1214 (4.3) | 512 (3.1) | 18 (0.4) | 49 (0.9) | 13 (0.4) | 31 (1.3) | 24 (1.2) | 11 (1.2) |
| Antipsychotics | 479 (2.2) | 493 (1.8) | 290 (1.8) | 93 (2.1) | 95 (1.7) | 69 (2.3) | 71 (2.9) | 50 (2.5) | 31 (3.3) |

Abbreviations: SD, standard deviation.

## Table S5: Baseline characteristics in missing and no missing. All statistics reported as number (% of column total), unless stated otherwise.

|  | **Missing** | **No missing** |
| --- | --- | --- |
| **Sample** | 91116 | 81629 |
| **Treatment group** |  |  |
| SUs | 31186 (34.2) | 26531 (32.5) |
| DPP4is | 38243 (42.0) | 35118 (43.0) |
| SGLT2is | 21687 (23.8) | 19980 (24.5) |
| **Age, years (SD)** | 57.9 (12.4) | 57.9 (12.3) |
| **Sex** |  |  |
| Male | 52348 (57.5) | 46731 (57.2) |
| Female | 38768 (42.5) | 34898 (42.8) |
| **Ethnicity** |  |  |
| White | 65853 (72.3) | 60695 (74.4) |
| South Asian | 12966 (14.2) | 12127 (14.9) |
| Black | 5430 (6.0) | 5022 (6.2) |
| Other | 1704 (1.9) | 1577 (1.9) |
| Mixed | 1001 (1.1) | 914 (1.1) |
| Not stated | 1453 (1.6) | 1294 (1.6) |
| **IMD level** |  |  |
| 1 (least deprived) | 14050 (15.4) | 12010 (14.7) |
| 2 | 16100 (17.7) | 14186 (17.4) |
| 3 | 17479 (19.2) | 15598 (19.1) |
| 4 | 21080 (23.1) | 19216 (23.5) |
| 5 (most deprived) | 22402 (24.6) | 20619 (25.3) |
| **Region of England** |  |  |
| North East | 2869 (3.1) | 2719 (3.3) |
| North West | 16291 (17.9) | 15106 (18.5) |
| Yorkshire And The Humber | 3071 (3.4) | 2823 (3.5) |
| East Midlands | 2003 (2.2) | 1810 (2.2) |
| West Midlands | 15368 (16.9) | 14252 (17.5) |
| East of England | 3597 (3.9) | 3327 (4.1) |
| South West | 19963 (21.9) | 18591 (22.8) |
| South Central | 17801 (19.5) | 13820 (16.9) |
| London | 10153 (11.1) | 9181 (11.2) |
| **Calendar year of baseline** | 2869 (3.1) | 2725 (3.3) |
| 2015 | 16291 (17.9) | 15168 (18.5) |
| 2016 | 3071 (3.4) | 2829 (3.5) |
| 2017 | 2003 (2.2) | 1813 (2.2) |
| 2018 | 15368 (16.9) | 14302 (17.4) |
| 2019 | 3597 (3.9) | 3332 (4.1) |
| 2020 | 19963 (21.9) | 18688 (22.8) |
| 2021 | 17801 (19.5) | 13902 (17.0) |
| 2022 | 10153 (11.1) | 9203 (11.2) |
| **Type 2 diabetes duration, years (SD)** | 5.0 (4.4) | 5.1 (4.3) |
| **HbA1c level** |  |  |
| <53 mmol/mol | 3640 (4.0) | 3335 (4.1) |
| 53-86 mmol/mol | 47828 (52.5) | 44464 (54.5) |
| >86 mmol/mol | 38193 (41.9) | 33830 (41.4) |
| **BMI Category** |  |  |
| Underweight | 187 (0.2) | 163 (0.2) |
| Normal | 6784 (7.4) | 6154 (7.5) |
| Overweight | 24086 (26.4) | 22241 (27.2) |
| Obese | 57074 (62.6) | 53071 (65.0) |
| **eGFR** |  |  |
| ≥60 mL/min/1.73 m² | 82519 (90.6) | 76983 (94.3) |
| <60 mL/min/1.73 m² | 5051 (5.5) | 4646 (5.7) |
| **History of alcohol misuse** |  |  |
| No | 84428 (92.7) | 75459 (92.4) |
| Yes | 6688 (7.3) | 6170 (7.6) |
| **Smoking status** |  |  |
| Never | 21817 (23.9) | 19127 (23.4) |
| Current | 24463 (26.8) | 21963 (26.9) |
| Former | 44825 (49.2) | 40539 (49.7) |
| **Healthcare utilization** |  |  |
| ≤10 consultation days | 46114 (50.6) | 40717 (49.9) |
| >10 consultation days | 45002 (49.4) | 40912 (50.1) |
| **Comorbidities** |  |  |
| Atrial fibrillation | 2339 (2.6) | 2081 (2.5) |
| Peripheral artery disease | 1013 (1.1) | 912 (1.1) |
| Hypertension | 47258 (51.9) | 43053 (52.7) |
| Neuropathy | 3500 (3.8) | 3185 (3.9) |
| Retinopathy | 13280 (14.6) | 12144 (14.9) |
| All cancers | 8757 (9.6) | 7786 (9.5) |
| Chronic liver disease | 774 (0.8) | 690 (0.8) |
| Chronic respiratory diseases | 5237 (5.7) | 4786 (5.9) |
| Rheumatoid arthritis | 1275 (1.4) | 1158 (1.4) |
| Dementia | 1028 (1.1) | 868 (1.1) |
| Severe mental illness | 2264 (2.5) | 2062 (2.5) |
| Common mental disorders | 27681 (30.4) | 24861 (30.5) |
| **Medications** |  |  |
| Ace inhibitors | 32183 (35.3) | 29328 (35.9) |
| Angiotensin II receptor blockers | 11841 (13.0) | 10801 (13.2) |
| Calcium channel blockers | 25049 (27.5) | 22852 (28.0) |
| Diuretics | 4370 (4.8) | 3888 (4.8) |
| Statins | 62076 (68.1) | 56910 (69.7) |
| Antiplatelets | 7481 (8.2) | 6743 (8.3) |
| Anticoagulants | 2878 (3.2) | 2550 (3.1) |
| Antipsychotics | 1794 (2.0) | 1627 (2.0) |

## Table S6: Crude incidence rates by treatment group and ethnic group for each outcome

1. **MACE**

|  | **Number of events, N** | **Median follow up time, years (IQR)** | **Total person time, years** | **Incidence rate, events per 1000 PYs** |
| --- | --- | --- | --- | --- |
| **Overall SU initiators** | **1859** | **3.5 (1.5-5.4)** | **108862.96** | **17.08** |
| White SU initiators | 1399 | 3.6 (1.6-5.5) | 76516.72 | 18.28 |
| South Asian SU initiators | 157 | 3.5 (1.5-5.5) | 15587.35 | 10.07 |
| Black SU initiators | 107 | 3.1 (1.4-5.1) | 8047.77 | 13.3 |
| **Overall DPP4i initiators** | **1942** | **3.1 (1.5-4.7)** | **122856.21** | **15.81** |
| White DPP4i initiators | 1554 | 3.2 (1.6-4.8) | 91755.96 | 16.94 |
| South Asian DPP4i initiators | 162 | 2.9 (1.5-4.5) | 17031.66 | 9.51 |
| Black DPP4i initiators | 55 | 3.0 (1.3-4.5) | 6156.83 | 8.93 |
| **Overall SGLT2i initiators** | **473** | **2.0 (0.8-3.5)** | **50924.04** | **9.29** |
| White SGLT2i initiators | 377 | 2.1 (0.9-3.6) | 39243.95 | 9.61 |
| South Asian SGLT2i initiators | 40 | 1.9 (0.7-3.2) | 6476.49 | 6.18 |
| Black SGLT2i initiators | 10 | 1.5 (0.7-3.1) | 1954.99 | 5.12 |
| **TOTAL** | **4274** | **2.9 (1.3-4.7)** | **282643.22** | **15.12** |

1. **Myocardial infarction**

|  | **Number of events, N** | **Median follow up time, years (IQR)** | **Total person time, years** | **Incidence rate, events per 1000 PYs** |
| --- | --- | --- | --- | --- |
| **Overall SU initiators** | **554** | **3.6 (1.6-5.5)** | **110658.5** | **5.01** |
| White SU initiators | 425 | 3.7 (1.6-5.6) | 77900.68 | 5.46 |
| South Asian SU initiators | 68 | 3.5 (1.6-5.5) | 15753.17 | 4.32 |
| Black SU initiators | 21 | 3.2 (1.4-5.2) | 8154.58 | 2.58 |
| **Overall DPP4i initiators** | **527** | **3.2 (1.6-4.8)** | **124673.73** | **4.23** |
| White DPP4i initiators | 414 | 3.3 (1.7-4.9) | 93234.54 | 4.44 |
| South Asian DPP4i initiators | 71 | 3.0 (1.5-4.6) | 17167.56 | 4.14 |
| Black DPP4i initiators | 11 | 3.0 (1.4-4.5) | 6222.92 | 1.77 |
| **Overall SGLT2i initiators** | **168** | **2.0 (0.8-3.6)** | **51288.92** | **3.28** |
| White SGLT2i initiators | 131 | 2.1 (0.9-3.7) | 39539.49 | 3.31 |
| South Asian SGLT2i initiators | 20 | 1.9 (0.7-3.2) | 6512.16 | 3.07 |
| Black SGLT2i initiators | 1 | 1.5 (0.7-3.1) | 1968.03 | .51 |
| **TOTAL** | **1249** | **3.0 (1.3-4.8)** | **286621.16** | **4.36** |

1. **Stroke**

|  | **Number of events, N** | **Median follow up time, years (IQR)** | **Total person time, years** | **Incidence rate, events per 1000 PYs** |
| --- | --- | --- | --- | --- |
| **Overall SU initiators** | **566** | **3.6 (1.6-5.5)** | **110793.** | **5.11** |
| White SU initiators | 424 | 3.7 (1.7-5.6) | 78030.44 | 5.43 |
| South Asian SU initiators | 53 | 3.6 (1.6-5.5) | 15777.75 | 3.36 |
| Black SU initiators | 34 | 3.2 (1.4-5.2) | 8132.85 | 4.18 |
| **Overall DPP4i initiators** | **540** | **3.2 (1.6-4.8)** | **124776.93** | **4.33** |
| White DPP4i initiators | 412 | 3.3 (1.7-4.9) | 93370.08 | 4.41 |
| South Asian DPP4i initiators | 50 | 3.0 (1.5-4.6) | 17193.9 | 2.91 |
| Black DPP4i initiators | 28 | 3.0 (1.4-4.5) | 6199.45 | 4.52 |
| **Overall SGLT2i initiators** | **120** | **2.0 (0.8-3.6)** | **51343.51** | **2.34** |
| White SGLT2i initiators | 95 | 2.1 (0.9-3.7) | 39582.58 | 2.4 |
| South Asian SGLT2i initiators | 8 | 1.9 (0.7-3.2) | 6530.24 | 1.23 |
| Black SGLT2i initiators | 4 | 1.5 (0.7-3.1) | 1955.75 | 2.05 |
| **TOTAL** | **1226** | **3.0 (1.3-4.8)** | **286913.45** | **4.27** |

1. **Heart Failure hospitalisation**

|  | **Number of events, N** | **Median follow up time, years (IQR)** | **Total person time, years** | **Incidence rate, events per 1000 PYs** |
| --- | --- | --- | --- | --- |
| **Overall SU initiators** | **806** | **3.6 (1.6-5.5)** | **110604.8** | **7.29** |
| White SU initiators | 625 | 3.7 (1.6-5.6) | 77873.43 | 8.03 |
| South Asian SU initiators | 50 | 3.6 (1.6-5.5) | 15784.88 | 3.17 |
| Black SU initiators | 51 | 3.2 (1.4-5.2) | 8127.7 | 6.27 |
| **Overall DPP4i initiators** | **928** | **3.2 (1.6-4.8)** | **124377.21** | **7.46** |
| White DPP4i initiators | 766 | 3.3 (1.7-4.9) | 92968.53 | 8.24 |
| South Asian DPP4i initiators | 61 | 3.0 (1.5-4.6) | 17185.3 | 3.55 |
| Black DPP4i initiators | 21 | 3.0 (1.4-4.5) | 6212.49 | 3.38 |
| **Overall SGLT2i initiators** | **184** | **2.0 (0.8-3.6)** | **51317.33** | **3.59** |
| White SGLT2i initiators | 154 | 2.1 (0.9-3.7) | 39562.56 | 3.89 |
| South Asian SGLT2i initiators | 15 | 1.9 (0.7-3.2) | 6509.45 | 2.3 |
| Black SGLT2i initiators | 2 | 1.5 (0.7-3.1) | 1967.51 | 1.02 |
| **TOTAL** | **1918** | **3.0 (1.3-4.8)** | **286299.35** | **6.7** |

1. **Cardiovascular death**

|  | **Number of events, N** | **Median follow up time, years (IQR)** | **Total person time, years** | **Incidence rate, events per 1000 PYs** |
| --- | --- | --- | --- | --- |
| **Overall SU initiators** | **376** | **3.7 (1.6-5.5)** | **111780.72** | **3.36** |
| White SU initiators | 262 | 3.7 (1.7-5.6) | 78787.52 | 3.33 |
| South Asian SU initiators | 22 | 3.6 (1.6-5.6) | 15885.14 | 1.38 |
| Black SU initiators | 19 | 3.2 (1.4-5.2) | 8190.69 | 2.32 |
| **Overall DPP4i initiators** | **379** | **3.2 (1.6-4.8)** | **125647.04** | **3.02** |
| White DPP4i initiators | 297 | 3.4 (1.7-4.9) | 94033.75 | 3.16 |
| South Asian DPP4i initiators | 22 | 3.0 (1.5-4.6) | 17277.16 | 1.27 |
| Black DPP4i initiators | 8 | 3.0 (1.4-4.6) | 6243.22 | 1.28 |
| **Overall SGLT2i initiators** | **86** | **2.1 (0.8-3.6)** | **51542.51** | **1.67** |
| White SGLT2i initiators | 68 | 2.1 (0.9-3.7) | 39745.54 | 1.71 |
| South Asian SGLT2i initiators | 3 | 1.9 (0.7-3.2) | 6541.38 | .46 |
| Black SGLT2i initiators | 3 | 1.5 (0.7-3.1) | 1968.15 | 1.52 |
| **TOTAL** | **841** | **3.0 (1.3-4.8)** | **288970.27** | **2.91** |

Abbreviations: MACE, major adverse cardiovascular events (a composite outcome of myocardial infarction, stroke, heart failure hospitalisation and cardiovascular death); IQR, interquartile range; PY, person year.

## Table S7: Hazard ratios and 95% confidence intervals for the interaction between ethnicity and medication class on risk of cardiovascular outcomes

1. **MACE**

|  | **White** | **South Asian** | **Black** | **TOTAL** |
| --- | --- | --- | --- | --- |
| **DPP4i vs SU** | p-interaction=0.12 | | | |
| Unadjusted | 0.93 (0.87-1.00) | 0.95 (0.76-1.18) | 0.66 (0.48-0.92) | 0.94 (0.88-1.00) |
| Partially adjusted | 0.85 (0.79-0.92) | 0.90 (0.72-1.12) | 0.62 (0.44-0.86) | 0.85 (0.79-0.91) |
| Adjusted | 0.91 (0.84-0.98) | 0.93 (0.75-1.16) | 0.64 (0.46-0.89) | 0.90 (0.84-0.96) |
| **SGLT2i vs SU** | p-interaction=0.38 | | | |
| Unadjusted | 0.54 (0.49-0.61) | 0.66 (0.46-0.93) | 0.39 (0.21-0.75) | 0.56 (0.51-0.62) |
| Partially adjusted | 0.76 (0.68-0.85) | 0.82 (0.58-1.16) | 0.51 (0.27-0.98) | 0.75 (0.68-0.84) |
| Adjusted | 0.79 (0.70-0.89) | 0.86 (0.61-1.22) | 0.51 (0.27-0.97) | 0.78 (0.70-0.87) |
| **SGLT2i vs DPP4i** | p-interaction=0.92 | | | |
| Unadjusted | 0.58 (0.52-0.65) | 0.69 (0.49-0.98) | 0.60 (0.31-1.17) | 0.60 (0.54-0.66) |
| Partially adjusted | 0.89 (0.80-1.00) | 0.91 (0.64-1.28) | 0.83 (0.43-1.62) | 0.89 (0.80-0.98) |
| Adjusted | 0.87 (0.78-0.98) | 0.93 (0.66-1.30) | 0.80 (0.41-1.57) | 0.87 (0.79-0.97) |

1. **Myocardial infarction**

|  | **White** | **South Asian** | **Black** | **TOTAL** |
| --- | --- | --- | --- | --- |
| **DPP4i vs SU** | p-interaction=0.63 | | | |
| Unadjusted | 0.83 (0.73-0.95) | 0.95 (0.69-1.33) | 0.68 (0.33-1.42) | 0.85 (0.76-0.96) |
| Partially adjusted | 0.78 (0.68-0.90) | 0.93 (0.66-1.29) | 0.67 (0.32-1.39) | 0.79 (0.70-0.90) |
| Adjusted | 0.86 (0.74-0.99) | 0.99 (0.71-1.38) | 0.71 (0.34-1.48) | 0.86 (0.76-0.98) |
| **SGLT2i vs SU** | p-interaction=0.51 | | | |
| Unadjusted | 0.64 (0.53-0.78) | 0.75 (0.45-1.24) | 0.22 (0.03-1.66) | 0.67 (0.56-0.80) |
| Partially adjusted | 0.78 (0.64-0.95) | 0.86 (0.52-1.43) | 0.27 (0.04-2.02) | 0.78 (0.65-0.94) |
| Adjusted | 0.89 (0.73-1.09) | 0.94 (0.57-1.56) | 0.28 (0.04-2.10) | 0.89 (0.73-1.07) |
| **SGLT2i vs DPP4i** | p-interaction=0.62 | | | |
| Unadjusted | 0.77 (0.64-0.94) | 0.79 (0.48-1.30) | 0.33 (0.04-2.51) | 0.79 (0.66-0.93) |
| Partially adjusted | 1.00 (0.82-1.22) | 0.93 (0.56-1.54) | 0.40 (0.05-3.09) | 0.99 (0.82-1.18) |
| Adjusted | 1.04 (0.85-1.27) | 0.95 (0.57-1.57) | 0.40 (0.05-3.04) | 1.03 (0.85-1.23) |

1. **Stroke**

|  | **White** | **South Asian** | **Black** | **TOTAL** |
| --- | --- | --- | --- | --- |
| **DPP4i vs SU** | p-interaction=0.72 | | | |
| Unadjusted | 0.82 (0.72-0.94) | 0.85 (0.58-1.26) | 1.06 (0.64-1.75) | 0.85 (0.76-0.96) |
| Partially adjusted | 0.76 (0.66-0.87) | 0.80 (0.54-1.19) | 0.97 (0.58-1.60) | 0.78 (0.69-0.88) |
| Adjusted | 0.83 (0.72-0.95) | 0.86 (0.58-1.28) | 1.03 (0.62-1.70) | 0.85 (0.75-0.96) |
| **SGLT2i vs SU** | p-interaction=0.84 | | | |
| Unadjusted | 0.46 (0.37-0.57) | 0.42 (0.20-0.86) | 0.49 (0.18-1.38) | 0.47 (0.38-0.57) |
| Partially adjusted | 0.64 (0.51-0.81) | 0.52 (0.25-1.07) | 0.62 (0.22-1.75) | 0.63 (0.52-0.78) |
| Adjusted | 0.71 (0.56-0.89) | 0.57 (0.28-1.17) | 0.66 (0.23-1.86) | 0.70 (0.57-0.86) |
| **SGLT2i vs DPP4i** | p-interaction=0.71 | | | |
| Unadjusted | 0.55 (0.45-0.69) | 0.49 (0.24-1.01) | 0.47 (0.16-1.33) | 0.55 (0.45-0.67) |
| Partially adjusted | 0.85 (0.68-1.07) | 0.65 (0.31-1.33) | 0.64 (0.22-1.84) | 0.82 (0.67-1.00) |
| Adjusted | 0.86 (0.69-1.08) | 0.66 (0.32-1.37) | 0.64 (0.22-1.84) | 0.83 (0.67-1.01) |

1. **Heart failure hospitalisation**

|  | **White** | **South Asian** | **Black** | **TOTAL** |
| --- | --- | --- | --- | --- |
| **DPP4i vs SU** | p-interaction=0.05 | | | |
| Unadjusted | 1.04 (0.94-1.15) | 1.14 (0.78-1.66) | 0.54 (0.33-0.90) | 1.05 (0.96-1.15) |
| Partially adjusted | 0.93 (0.83-1.03) | 1.06 (0.73-1.55) | 0.49 (0.29-0.82) | 0.92 (0.83-1.01) |
| Adjusted | 0.95 (0.85-1.06) | 1.04 (0.71-1.52) | 0.50 (0.30-0.84) | 0.94 (0.85-1.04) |
| **SGLT2i vs SU** | p-interaction=0.14 | | | |
| Unadjusted | 0.51 (0.43-0.60) | 0.79 (0.45-1.41) | 0.18 (0.04-0.73) | 0.52 (0.45-0.61) |
| Partially adjusted | 0.79 (0.66-0.94) | 1.07 (0.60-1.90) | 0.25 (0.06-1.05) | 0.77 (0.65-0.92) |
| Adjusted | 0.76 (0.64-0.92) | 1.06 (0.60-1.89) | 0.24 (0.06-0.98) | 0.75 (0.63-0.89) |
| **SGLT2i vs DPP4i** | p-interaction=0.55 | | | |
| Unadjusted | 0.49 (0.41-0.58) | 0.70 (0.40-1.22) | 0.33 (0.08-1.38) | 0.50 (0.43-0.58) |
| Partially adjusted | 0.85 (0.71-1.01) | 1.01 (0.58-1.76) | 0.52 (0.12-2.19) | 0.84 (0.71-0.99) |
| Adjusted | 0.80 (0.67-0.96) | 1.02 (0.58-1.79) | 0.47 (0.11-2.00) | 0.80 (0.68-0.94) |

1. **Cardiovascular death**

|  | **White** | **South Asian** | **Black** | **TOTAL** |
| --- | --- | --- | --- | --- |
| **DPP4i vs SU** | p-interaction=0.30 | | | |
| Unadjusted | 0.93 (0.80-1.09) | 0.89 (0.50-1.59) | 0.50 (0.22-1.15) | 0.92 (0.80-1.06) |
| Partially adjusted | 0.86 (0.73-1.01) | 0.84 (0.47-1.50) | 0.46 (0.20-1.06) | 0.83 (0.72-0.97) |
| Adjusted | 0.95 (0.80-1.12) | 0.91 (0.51-1.62) | 0.48 (0.21-1.10) | 0.91 (0.78-1.06) |
| **SGLT2i vs SU** | p-interaction=0.73 | | | |
| Unadjusted | 0.55 (0.42-0.70) | 0.36 (0.11-1.21) | 0.62 (0.18-2.11) | 0.54 (0.42-0.68) |
| Partially adjusted | 0.87 (0.67-1.13) | 0.49 (0.15-1.66) | 0.91 (0.27-3.07) | 0.81 (0.63-1.04) |
| Adjusted | 0.92 (0.70-1.20) | 0.56 (0.17-1.90) | 0.92 (0.27-3.12) | 0.86 (0.67-1.11) |
| **SGLT2i vs DPP4i** | p-interaction=0.46 | | | |
| Unadjusted | 0.58 (0.46-0.75) | 0.41 (0.12-1.32) | 1.25 (0.33-4.70) | 0.58 (0.46-0.74) |
| Partially adjusted | 1.01 (0.78-1.30) | 0.59 (0.18-1.92) | 1.97 (0.52-7.44) | 0.97 (0.76-1.24) |
| Adjusted | 0.97 (0.75-1.25) | 0.62 (0.19-2.03) | 1.92 (0.51-7.27) | 0.94 (0.74-1.21) |

* Hazard ratios (HRs) estimate risk of outcomes associated with comparator treatment vs reference treatment, with HRs <1 favouring the comparator and HRs >1 favouring the reference.

* Ethnicity added as a covariate to obtain total population estimates and an interaction term to obtain ethnic group-specific estimates.

* Partially adjusted models additionally adjusted for sex, age, index of multiple deprivation, duration of type 2 diabetes and region. Adjusted models additional adjusted for BMI, HbA1c, smoking, alcohol abuse, healthcare utilisation rate, pre-existing comorbidities and medication use in the year before baseline.

* P-interaction obtained using a Wald test for interaction between medication class and ethnicity in adjusted models

## Table S8. Sensitivity analysis: Hazard ratios and 95% confidence intervals for interaction between ethnicity and medication class on risk of MACE using complete case sample

|  | **White** | **South Asian** | **Black** | **TOTAL** |
| --- | --- | --- | --- | --- |
| **DPP4i vs SU** | p-interaction=0.08 | | | |
| Unadjusted | 0.94 (0.87-1.02) | 0.99 (0.79-1.25) | 0.65 (0.46-0.91) | 0.95 (0.89-1.02) |
| Partially adjusted | 0.87 (0.80-0.94) | 0.94 (0.75-1.18) | 0.61 (0.44-0.86) | 0.87 (0.81-0.93) |
| Adjusted | 0.93 (0.85-1.00) | 0.97 (0.77-1.22) | 0.63 (0.45-0.89) | 0.92 (0.85-0.98) |
| **SGLT2i vs SU** | p-interaction=0.42 | | | |
| Unadjusted | 0.53 (0.47-0.60) | 0.64 (0.44-0.91) | 0.40 (0.21-0.76) | 0.55 (0.49-0.61) |
| Partially adjusted | 0.74 (0.66-0.84) | 0.79 (0.55-1.14) | 0.51 (0.27-0.98) | 0.74 (0.66-0.83) |
| Adjusted | 0.78 (0.69-0.89) | 0.84 (0.58-1.21) | 0.51 (0.27-0.98) | 0.77 (0.68-0.87) |
| **SGLT2i vs DPP4i** | p-interaction=0.99 | | | |
| Unadjusted | 0.57 (0.50-0.64) | 0.64 (0.45-0.92) | 0.61 (0.31-1.21) | 0.58 (0.52-0.65) |
| Partially adjusted | 0.86 (0.76-0.97) | 0.84 (0.59-1.20) | 0.83 (0.42-1.64) | 0.85 (0.76-0.95) |
| Adjusted | 0.84 (0.75-0.95) | 0.86 (0.60-1.23) | 0.81 (0.41-1.59) | 0.84 (0.75-0.94) |

## Table S9. Sensitivity analysis: interaction between treatment class and ethnicity on risk of MACE using per-protocol exposure definition where follow-up was truncated after treatment stopping, switching or adding

|  | **White** | **South Asian** | **Black** | **TOTAL** |
| --- | --- | --- | --- | --- |
| **DPP4i vs SU** | p-interaction=0.45 | | | |
| Unadjusted | 0.88 (0.79-0.98) | 0.85 (0.57-1.25) | 0.62 (0.33-1.16) | 0.88 (0.80-0.98) |
| Partially adjusted | 0.80 (0.71-0.89) | 0.78 (0.53-1.16) | 0.56 (0.30-1.04) | 0.79 (0.71-0.88) |
| Adjusted | 0.85 (0.76-0.96) | 0.80 (0.54-1.19) | 0.58 (0.31-1.08) | 0.84 (0.75-0.94) |
| **SGLT2i vs SU** | p-interaction=0.75 | | | |
| Unadjusted | 0.42 (0.35-0.49) | 0.54 (0.30-0.98) | 0.32 (0.10-1.05) | 0.43 (0.37-0.50) |
| Partially adjusted | 0.61 (0.51-0.74) | 0.68 (0.37-1.22) | 0.42 (0.13-1.38) | 0.61 (0.51-0.72) |
| Adjusted | 0.64 (0.53-0.77) | 0.71 (0.39-1.29) | 0.42 (0.13-1.39) | 0.63 (0.53-0.75) |
| **SGLT2i vs DPP4i** | p-interaction=0.86 | | | |
| Unadjusted | 0.48 (0.40-0.56) | 0.64 (0.36-1.14) | 0.52 (0.15-1.75) | 0.48 (0.41-0.57) |
| Partially adjusted | 0.77 (0.65-0.92) | 0.87 (0.48-1.55) | 0.75 (0.22-2.57) | 0.77 (0.65-0.90) |
| Adjusted | 0.75 (0.63-0.89) | 0.89 (0.49-1.59) | 0.73 (0.22-2.50) | 0.75 (0.63-0.88) |

* Individuals followed from first prescription of second-line medication until end of registration, end of the practice contributing data to CPRD, end of the study period (April 2022), non-cardiovascular death, the occurrence of an outcome of interest, or adding, swapping or after a gap in prescription records of 60 days or more, whichever occurred first.

* Missing data handled using multiple imputation.

## Table S10. Sensitivity analysis: interaction between treatment class and ethnicity on risk of MACE using per-protocol exposure definition where follow-up was truncated after treatment stopping, switching or adding, and inverse probability of censoring weights (IPCW) applied to account for informative censoring

|  | **White** | **South Asian** | **Black** | **TOTAL** |
| --- | --- | --- | --- | --- |
| **DPP4i vs SU** | p-interaction=0.26 | | | |
| Adjusted | 0.75 (0.66-0.86) | 0.76 (0.49-1.19) | 0.43 (0.22-0.83) | 0.73 (0.65-0.84) |
| **SGLT2i vs SU** | p-interaction=0.62 | | | |
| Adjusted | 0.55 (0.45-0.68) | 0.71 (0.36-1.41) | 0.37 (0.11-1.26) | 0.54 (0.45-0.66) |
| **SGLT2i vs DPP4i** | p-interaction=0.75 | | | |
| Adjusted | 0.73 (0.60-0.89) | 0.94 (0.49-1.81) | 0.87 (0.25-3.05) | 0.74 (0.61-0.89) |

* Individuals followed from first prescription of second-line medication until end of registration, end of the practice contributing data to CPRD, end of the study period (April 2022), non-cardiovascular death, the occurrence of an outcome of interest, or adding, swapping or after a gap in prescription records of 60 days or more, whichever occurred first.

* Complete case sample used.

* Adjusted for all pre-specified confounders

## Table S11: Adjusted Cox failure curves for MACE by treatment group for each ethnic group

1. **Total study population**

1. **White population**

1. **South Asian population**

1. **Black population**

## References

1. NHS Data Model and Dictionary. PERSON STATED GENDER CODE: UK National Health Service; 2025 [Available from: https://www.datadictionary.nhs.uk/attributes/person_stated_gender_code.html.

2. Shiekh SI, Harley M, Ghosh RE, Ashworth M, Myles P, Booth HP, et al. Completeness, agreement, and representativeness of ethnicity recording in the United Kingdom’s Clinical Practice Research Datalink (CPRD) and linked Hospital Episode Statistics (HES). Popul Health Metr. 2023;21(1):3.

3. Mathur R, Bhaskaran K, Chaturvedi N, Leon DA, vanStaa T, Grundy E, et al. Completeness and usability of ethnicity data in UK-based primary care and hospital databases. Journal of public health. 2014;36(4):684-92.

4. Bhaskaran K, Forbes HJ, Douglas I, Leon DA, Smeeth L. Representativeness and optimal use of body mass index (BMI) in the UK Clinical Practice Research Datalink (CPRD). BMJ open. 2013;3(9):e003389.

5. NHS. Conditions: Obesity 2023 [Available from: https://www.nhs.uk/conditions/obesity/.

6. Levey AS, Stevens LA, Schmid CH, Zhang Y, Castro III AF, Feldman HI, et al. A new equation to estimate glomerular filtration rate. Annals of internal medicine. 2009;150(9):604-12.

7. Griffiths K, Gama RM, Fabian J, Molokhia M. Interpreting an estimated glomerular filtration rate (eGFR) in people of black ethnicities in the UK. bmj. 2023;380.

8. Chronic kidney disease: assessment and management [NG203]. National Insititute for Healthcare Excellence (NICE); 2021.
